# Identification of Pipeline Axial Tensile Stress Using Geometric and ACSM In-Line Inspection Data

**DOI:** 10.3390/s26185748

**Published:** 2026-09-10

**Authors:** Zhengqiang Lei, Guangyong Yang, Yanbing Wang, Rui Li

**Affiliations:** 1 PipeChina Institute of Science and Technology, Tianjin 300457, China; 2 College of Petroleum Engineering, China University of Petroleum (Beijing), Beijing 102249, China

**Keywords:** in-line inspection, ACSM, pipeline, stress, geometric caliper

## Abstract

Accurate identification of high-stress axial tensile sections in pipelines remains a significant challenge for stress in-line inspection (ILI) technology. To address this, we designed and conducted dynamic calibration tests for Alternating Current Stress Measurement (ACSM), alongside a pull test combining geometric and ACSM stress ILI sensors. Results demonstrate approximately linear correlation between applied axial stress and the mean ACSM signal, characterized by a distinct baseline downshift under tensile loading. However, irregular high-frequency fluctuations compromise quantitative accuracy, obscuring the distinction between actual stress variations and environmental noise. To mitigate this, a new analysis method is proposed, integrating geometric deformation data with ACSM signals. This approach effectively attributes signal variations to axial tensile deformation, significantly enhancing assessment reliability. Furthermore, we conducted an integrated analysis of ACSM and geometric ILI data for an in-service D813 oil pipeline. The synergistic variation between geometric deformations and ACSM signals was validated at pipe sections exhibiting localized stress alterations induced by dents and wall thickness transitions. By leveraging Poisson’s effect in conjunction with geometric ILI data, we identify and analyze ACSM stress concentration signals. Thereby, the adverse impacts of weld seams, mechanical noise, and other interferences in signal fidelity are mitigated. This study confirms that integrated analysis of geometric and ACSM ILI data is beneficial for high axial stress identification of in-service pipeline.

## 1. Introduction

Long-distance oil and gas pipelines constitute the arterial infrastructure of the global energy supply chain, as the International Energy Agency estimates that more than 2.5 million kilometers of pipelines are currently in service worldwide [1]. With the increasing application of large-diameter high-grade steel pipelines, the excessive load stress on pipelines caused by geological environment changes is an important reason for girth weld cracking failure [2]. Therefore, the effective detection and identification of axial stress in in-service pipelines have emerged as a research focus in pipeline safety and maintenance technology in recent years.

Alternating Current Stress Measurement (ACSM) is widely used for in-line inspection (ILI) due to its advantage of dynamic testing of pipeline stress as an electromagnetic non-destructive testing (NDT) method. ACSM exploits the inverse magnetostriction (Villari) effect—the stress-dependent change in magnetic permeability of ferromagnetic materials—together with the skin-depth-controlled penetration of alternating magnetic fields, to relate measurable flux density signals to the mechanical stress state of the pipe wall [3,4]. Since the work of Chen and Brennan in the late 1990s [5], ACSM has evolved from a laboratory curiosity into a field-deployable ILI tool, culminating in recent proprietary systems capable of biaxial stress measurement [6,7,8]. While the magnetomechanical response of pipeline steel is sensitive to microstructure, texture, prior plastic deformation history, and thermal treatment [3,9,10], for high-grade steels, the near-Villari reversal at high stress levels complicates signal interpretation [11]. Thus ILI tools usually combine ACSM with IMU providing a pathway for ILI-data-integrated analysis to improve the identification of bending stress and axial stress [12].

For in-service pipelines, under the effect of axial tensile stress, the pipeline elongates axially while simultaneously undergoing circumferential contraction due to the Poisson effect. Theoretically, this results in a corresponding reduction in the inner diameter. Using geometric and ACSM stress ILI could be beneficial for identification of pipeline tensile failure risk. In this study, we designed a full-scale pipeline-tensile-loading test device, and conducted geometry and ACSM ILI traction tests to verify the response of geometric and ACSM stress signals in tensile stress pipe sections. An integrated analysis method applicable to in-service pipelines, integrating geometry and ACSM stress ILI, was proposed to identify the risk of axial tensile fracture in pipelines.

## 2. Pull Test of Geometry and ACSM ILI Tools

### 2.1. Challenges in Dynamic Inspection of Pipeline Stress Using ACSM

**Figure 1:**
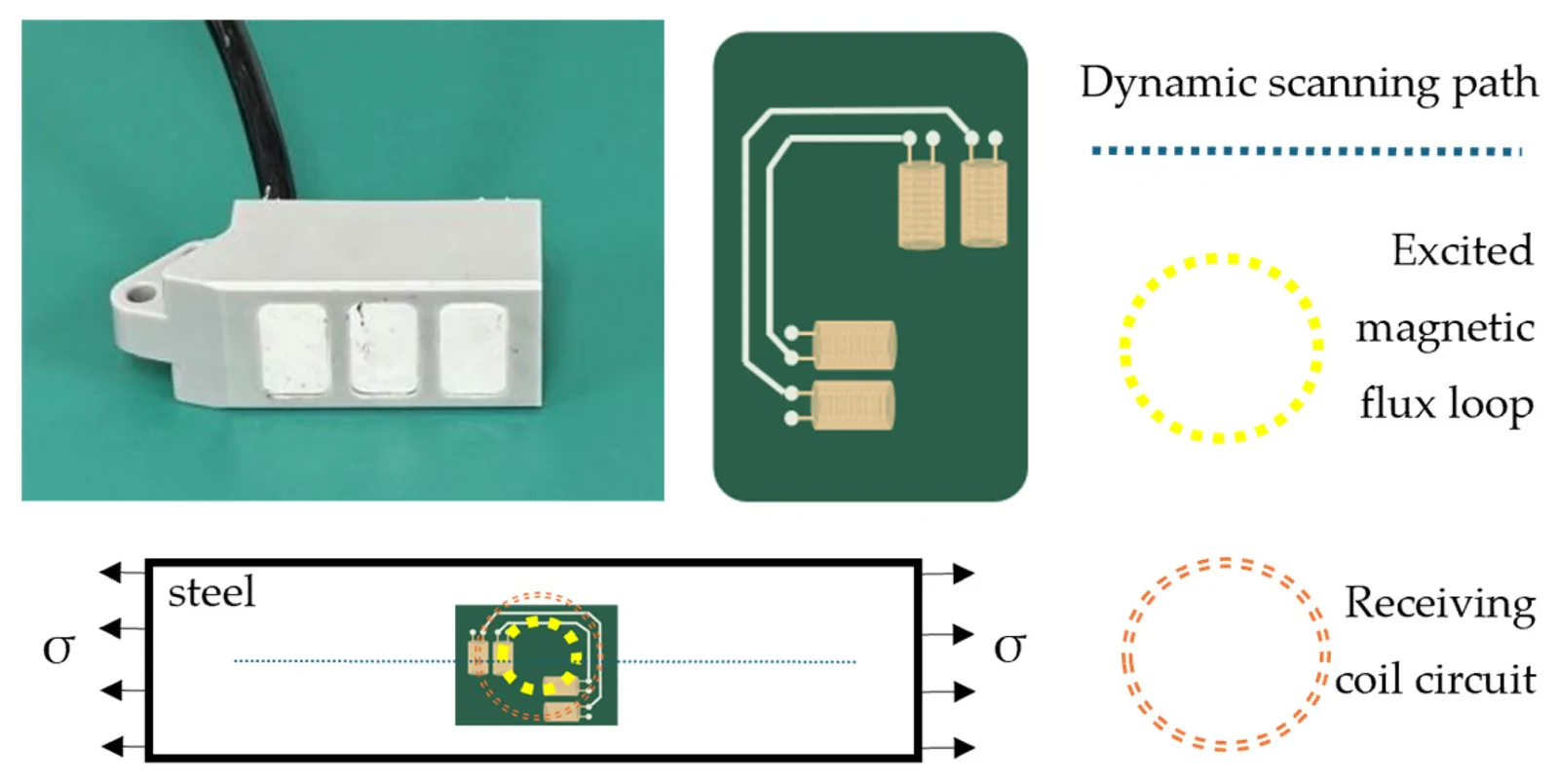
Picture of the ACSM sensor for stress scanning of pipe steel.

The ACSM sensors used in this study consists of two coil units and a magnetically conductive core as shown in Figure 1. Each coil unit contains one excitation coil and one receiving coil. The coils are wound with enameled copper wire with a diameter of 0.15 mm, each having 200 turns. The two coil units are placed orthogonally, with their excitation coils wound in the same direction and connected in series to receive identical excitation signals. The two detection coils are connected in series opposition to obtain a differential signal. Shielded cables are used for signal wiring, which exit centrally from the rear of the housing.

When no stress is applied to the ferromagnetic material, it exhibits magnetic isotropy. Since the two sets of coils are positioned at the same magnetic potential, the induced differential output signal from the detection coils is zero. However, when stress exists within the ferromagnetic material, stress-induced magnetic anisotropy alters the magnetic permeability along the directions of the two coils. This results in different impedances between the two detection coils, generating an induced voltage across them.

**Figure 2:**
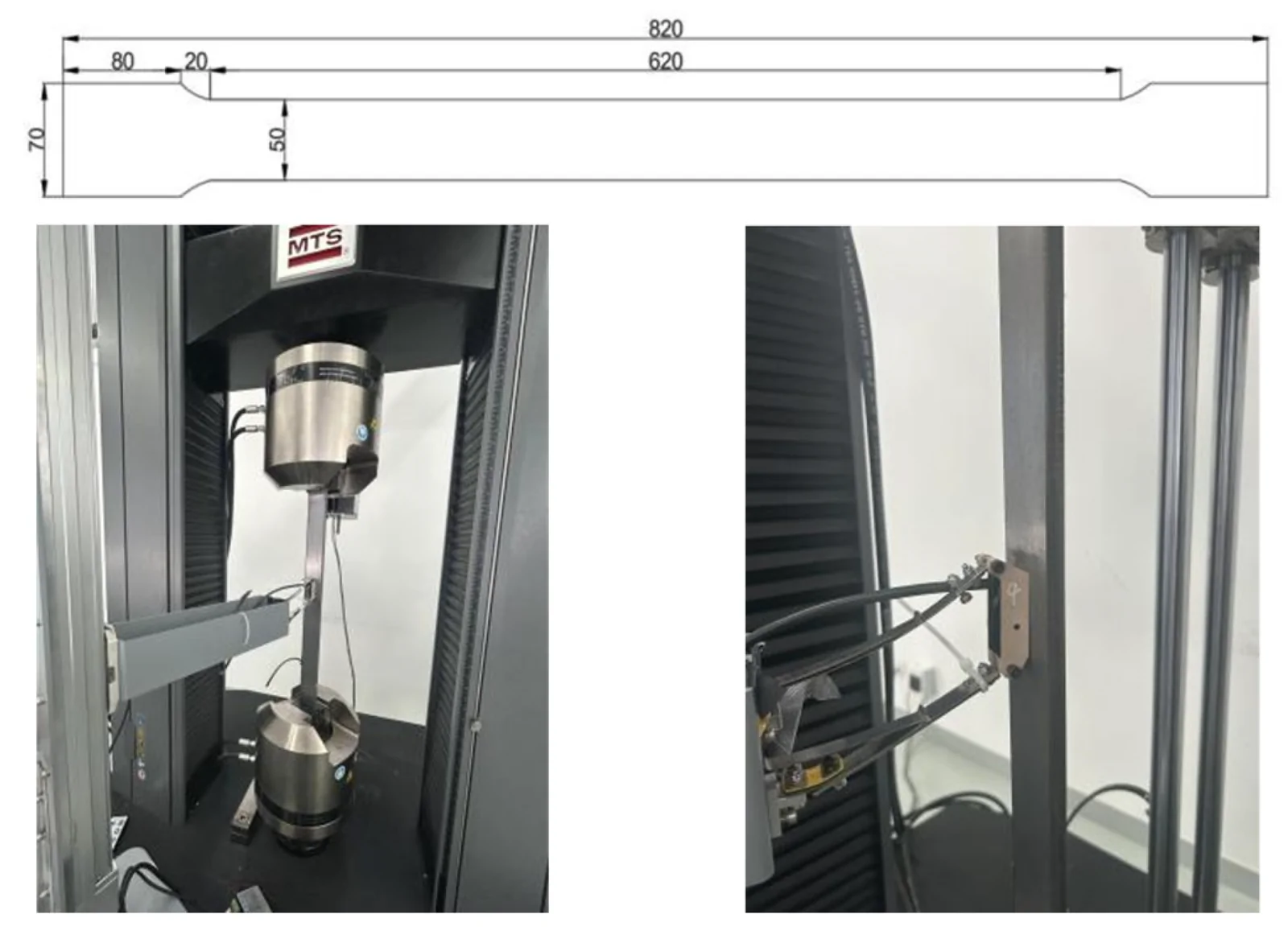
Dynamic calibration test of the ACSM sensor with X52 steel.

Figure 2 illustrates the experimental setup for the dynamic calibration test of the ACSM sensor utilizing X52 steel specimens. The left panel presents a tensile testing machine (MTS System Corporation, Eden Prairie, MN, USA) equipped with a dedicated dynamic test fixture. The fixture ensures the stability and consistent lift-off of the probe during dynamic testing with the moving speed of 0.1 m/s. The size of tensile specimen is shown in Figure 2 with the thickness of 10 mm. At each predetermined stress level during tensile loading, the ACSM probe scanned from top to bottom to acquire the corresponding ACSM signal data.

**Figure 3:**
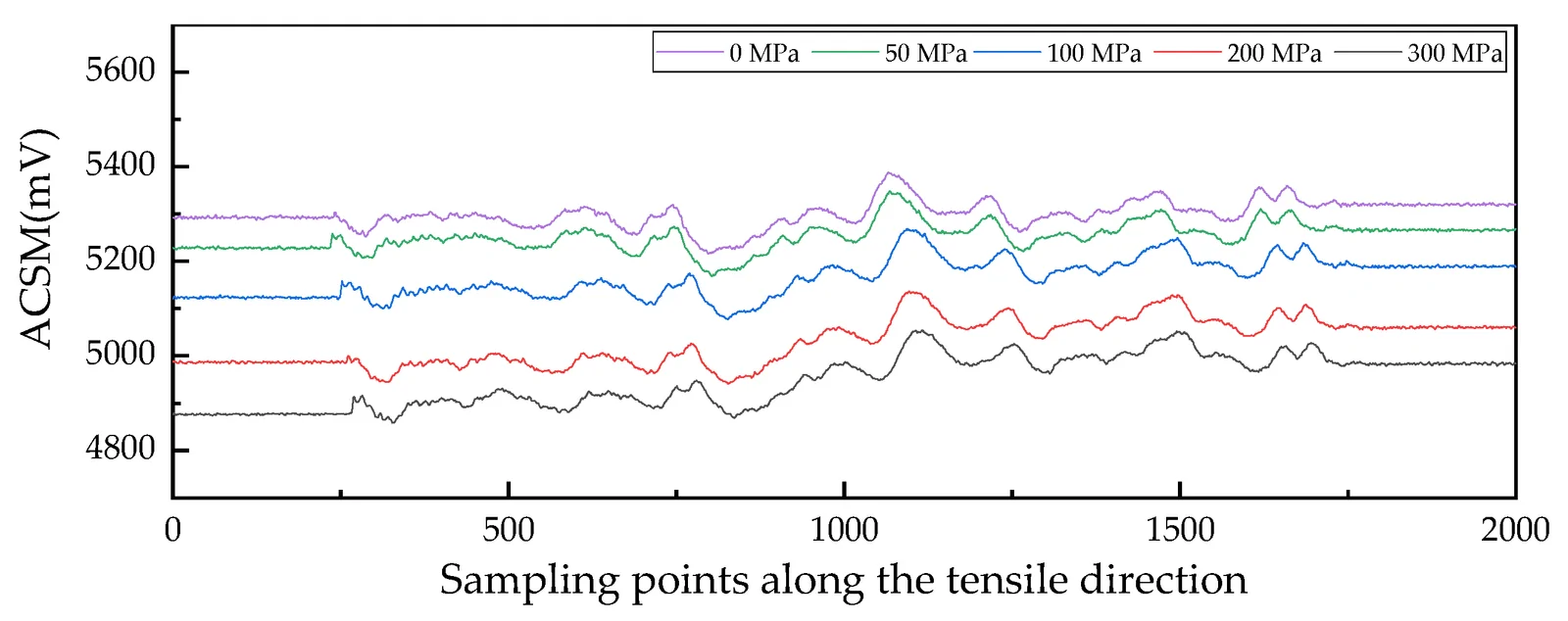
Dynamic calibration results of the ACSM sensor under different stress levels.

Figure 3 illustrates the ACSM signal acquired along the tensile direction of an X52 steel specimen subjected to stepwise increasing tensile loads. The horizontal axis represents the sampling points from top to bottom along the tensile direction, while the vertical axis denotes the ACSM signal intensity in millivolts (mVs). Each colored trace corresponds to a distinct applied stress level, as indicated in the legend ranging from 0 MPa to 300 MPa.

A clear trend of mean signal displacement is observed with increasing applied stress. Specifically, the output baseline exhibits an downward shift as the tensile load increases. For instance, at 0 MPa, the average signal hovers around 5300 mV, whereas at 300 MPa, it drops significantly to an average of approximately 4950 mV. This indicates a strong correlation between the applied axial stress and the mean ACSM signal shift.

**Figure 4:**
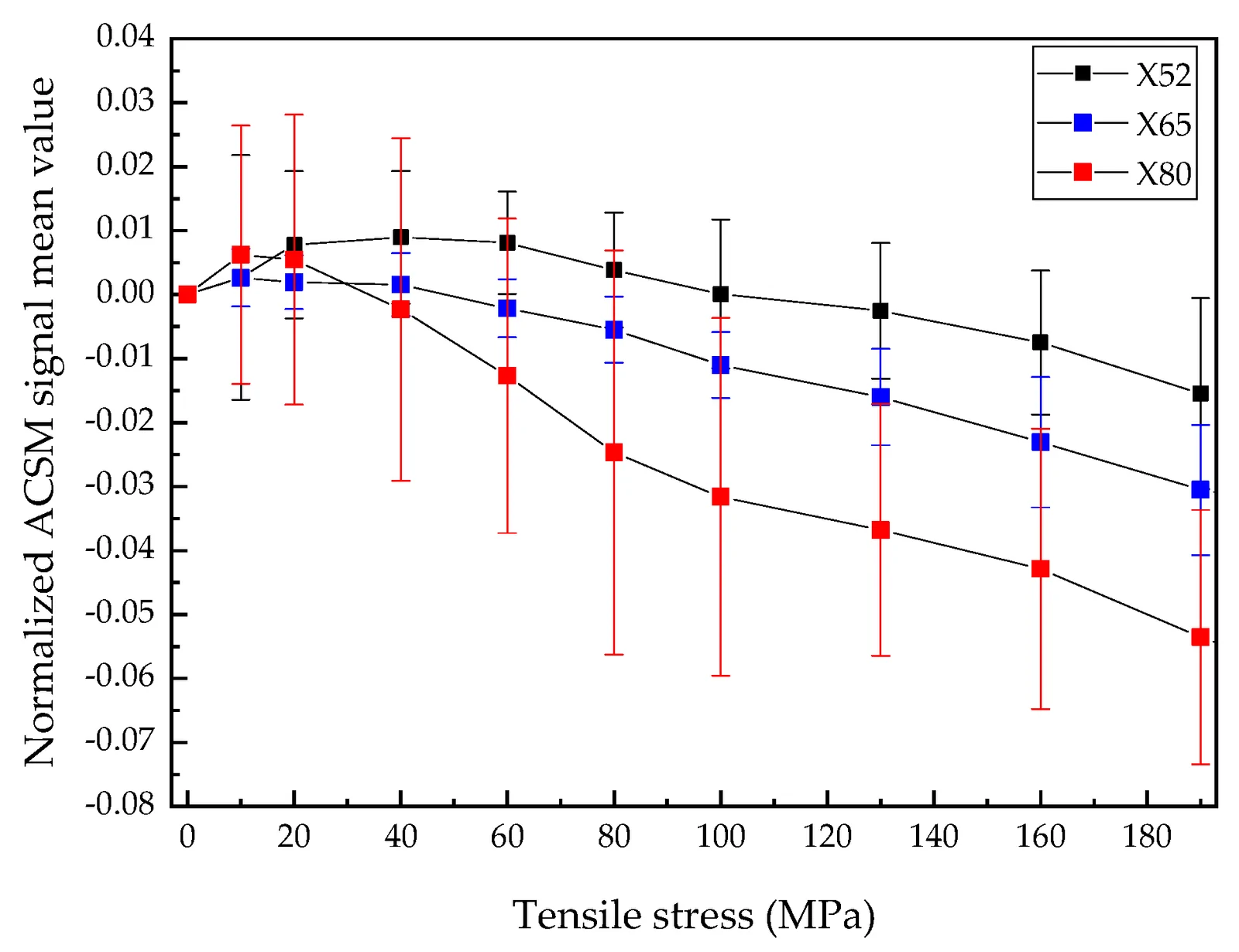
Plot of normalized ACSM signal as a function of stress for different steel grades.

Figure 4 illustrates the variation in the normalized ACSM signal mean value as a function of applied tensile stress for pipeline steels of different strength grades (X52, X65, and X80). Across the entire stress range investigated (0–190 MPa), all three steel grades exhibit a consistent trend of negative drift in the normalized signal with increasing stress, although the magnitude and rate of attenuation demonstrate distinct grade dependent characteristics. Specifically, the normalized ACSM signal for X52 remains relatively stable at low stress levels (approximately 0.00 to 0.01 between 0 and 20 MPa) before exhibiting a gradual linear decline to approximately −0.015 at 190 MPa. In contrast, the higher grade X65 and X80 steels display more pronounced negative responses, with X80 showing the steepest descent from near zero values to approximately −0.055 over the same stress interval. Furthermore, the vertical error bars indicate substantial scattering in the measurements, particularly at elevated stress levels and for the high strength X80 grade. Across the different steel grades examined, the normalized ACSM signal exhibits a consistent trend with increasing stress levels.

However, superimposed on the mean signal shift are high frequency fluctuations. These variations are inherent to the dynamic measurement process and may be attributed to microstructural heterogeneities, electromagnetic interference, or slight mechanical vibrations during the data acquisition window within the pipe steel.

Even at identical stress levels, the irregular fluctuations inherent in ACSM signals pose significant challenges to the quantitative accuracy of in-service pipeline stress ILI. For in-service pipeline ILI data, it is difficult to decouple these signal perturbations stem of actual alterations in the stress state from extraneous interferences. Consequently, integrating ILI data regarding geometric deformation can effectively resolve this ambiguity. By corroborating an increase in the ACSM stress signal with a simultaneous reduction in pipe radius, one can confirm that the signal variation is induced by tensile deformation of the pipeline. This multivariate data integration approach could substantially enhance the reliability and analytical accuracy of ACSM ILI.

### 2.2. Pull Test Design for Geometry and ACSM ILI Tools

**Figure 5:**
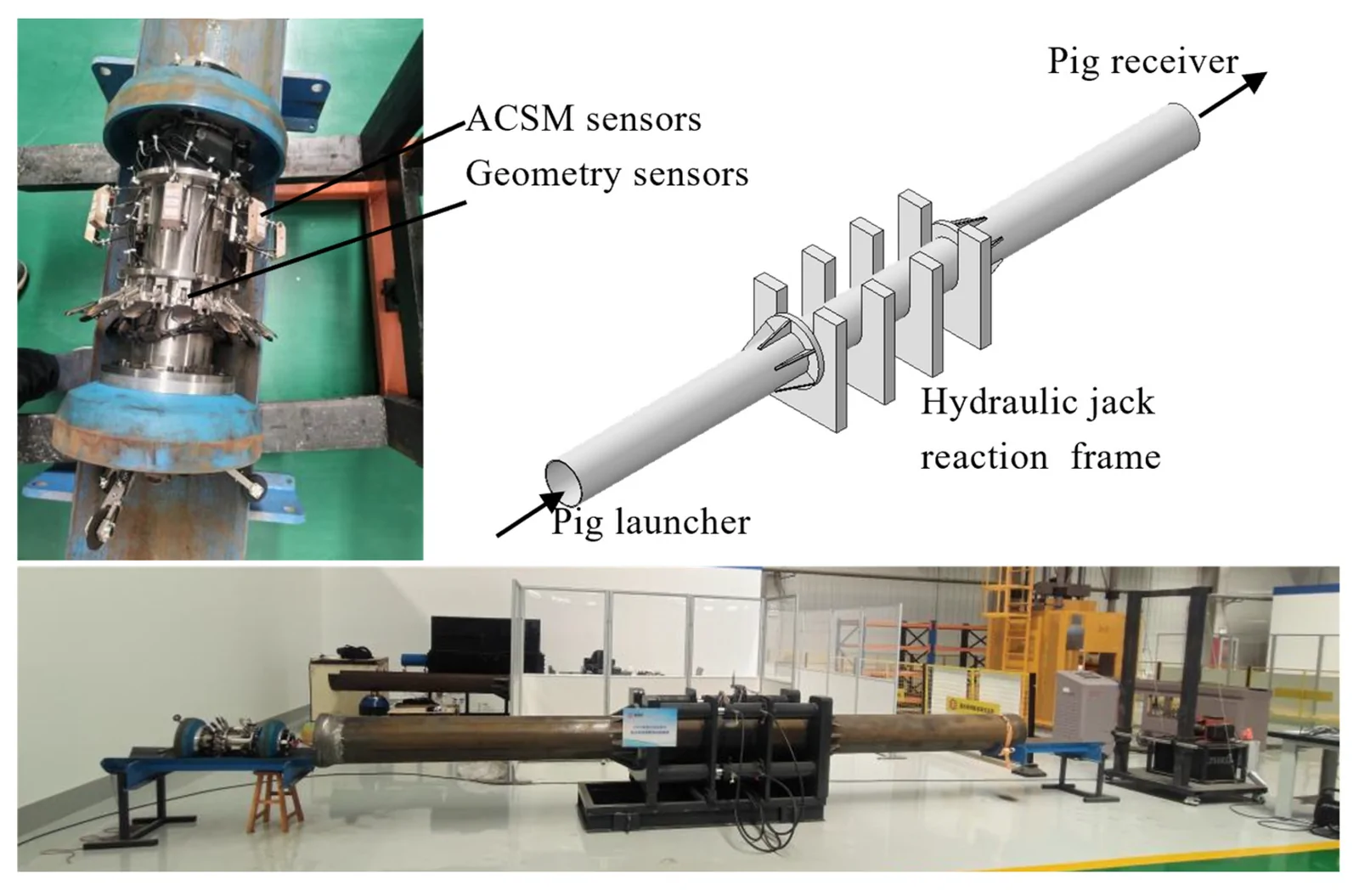
Geometry and ACSM ILI tools for the pull test of axial-tensile-loaded pipe.

The geometry and ACSM ILI tools used in the pull test is shown in Figure 5. The outer diameter of the X52 steel pipe is 355 mm with a thickness of 6 mm and a total length of 6 m. The pipe is tensile loaded by the flange through the hydraulic jack reaction frame, and the distance between the two flanges is nearly 1.6 m. The tensile loaded pipe section between the two flanges, in contrast to the unloaded sections at both ends, facilitates comparative analysis of pipeline geometry and ACSM stress ILI signal data.

The maximum tensile stress of the pipe produced by the frame is over 240 MPa. When the tool passes through the tensile section of the pipe, the ACSM probe detects an increase in stress, while the geometric probe identifies a reduction in the pipe’s inner diameter.

## 3. Results of Pull Test and Discussions

### 3.1. Geometric and ACSM Stress ILI Data Acquired by Pull Tests

The loading tests were conducted at four axial stress levels: 50 MPa, 100 MPa, 150 MPa, and 200 MPa. After the completion of loading at each stress level, a pull test was performed, during which geometric and ACSM stress ILI data were acquired.

**Figure 6:**
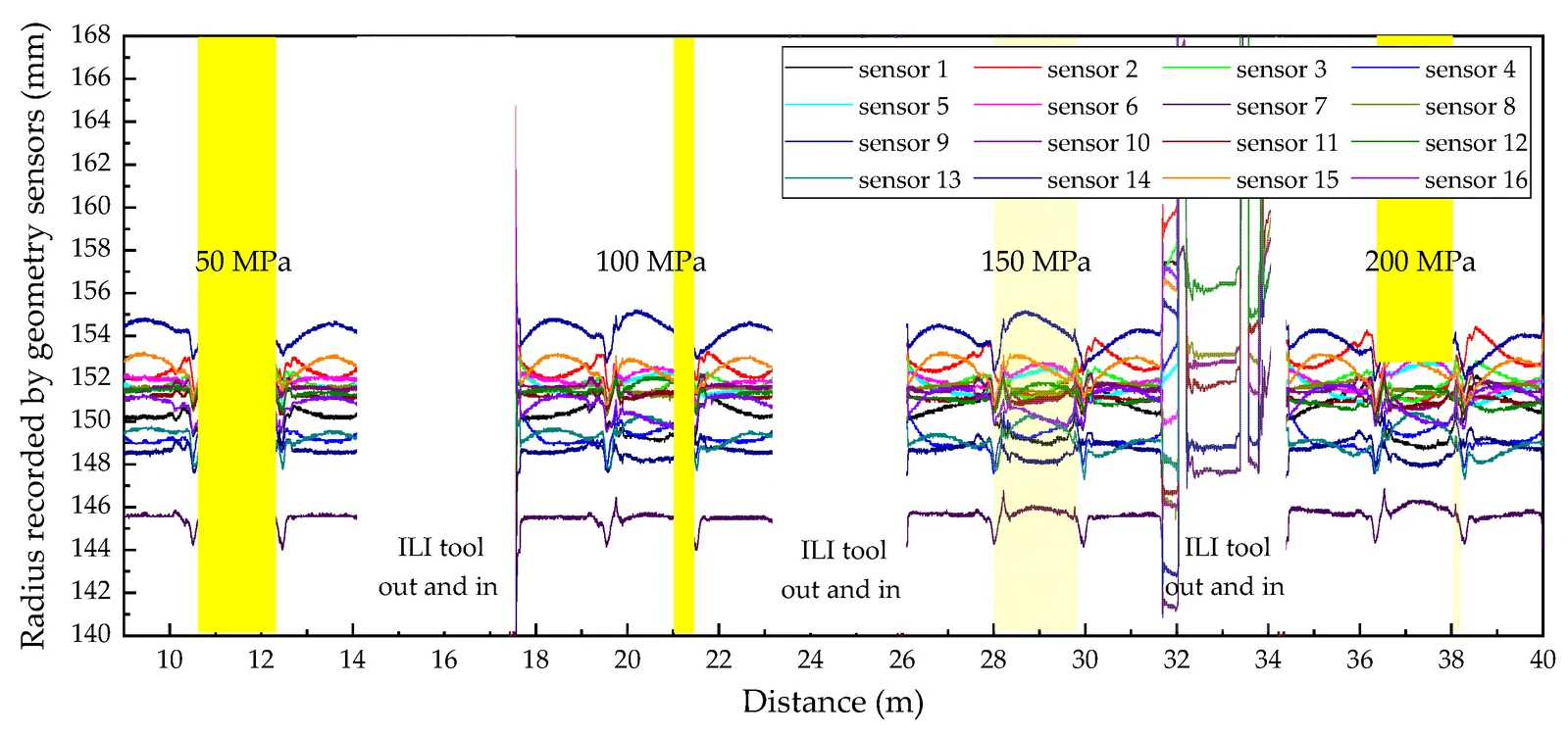
Pipe radius measured by geometry sensors through pull test under different axial stress.

Figure 6 shows the pipe radius measured by geometry sensors through pull test under the four axial stress levels. There are 16 geometry sensors in the ILI tool. Theoretically, as the axial tensile stress in the pipe increases, the radius of the tensioned section decreases. However, based on the radius measurement data from the pipeline geometry ILI data, the reduction in pipe radius is present but not significant.

**Figure 7:**
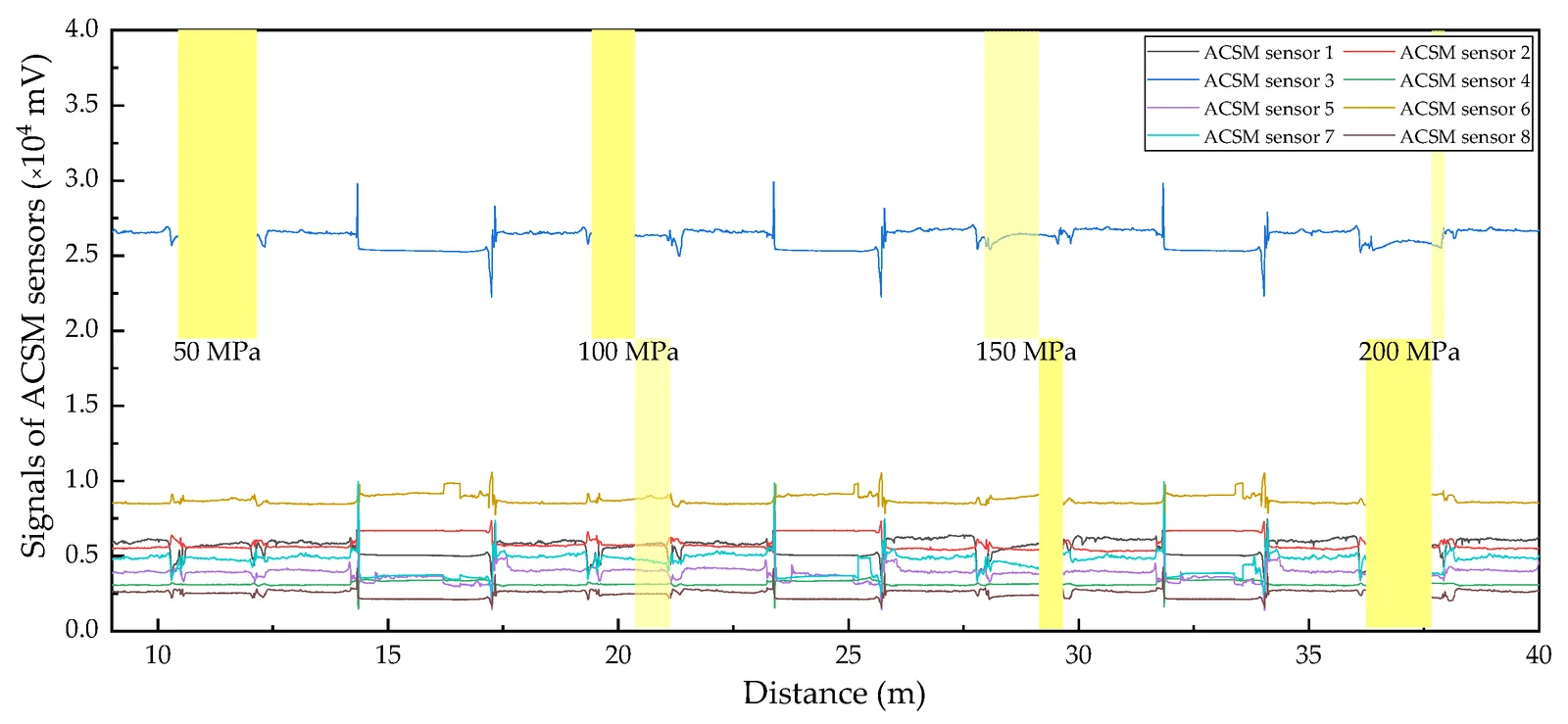
ACSM signals through pull test under different axial stress.

Figure 7 shows ACSM signal through pull test under the four axial stress levels. There are eight ACSM sensors in the ILI tool. Theoretically, as the axial tensile stress in the pipe increases, the radius of the tensile loaded pipe decreases. However, based on the radius measurement data from the pipeline geometry ILI data, the reduction in pipe radius is present but not significant. It can be observed that with the increase in axial tensile stress, the ACSM signals from the tensile loaded pipe exhibit certain variations. Moreover, the trends of these signal changes differ among individual ACSM sensors, which may be attributed to the asymmetrical distribution of axial tensile stress in the pipe cross section.

### 3.2. An Integrated Analysis Method Proposed to Identify HighTensileStress Region

Provided that the change in pipe wall thickness during tensile deformation is neglected, the reduction in the inner diameter (∆R) can be related to the axial strain of the pipe as shown in Equation (1).

(1) $$\frac{∆R}{R}=-v\cdot \varepsilon_{axial}$$

where *R* is the original radius of the pipe, *v* is the Poisson’s ratio, and $\varepsilon_{axial}$ is the axial strain. For tensile deformed pipe segments, geometric ILI data reveal a tendency toward reduced radius, while ACSM signals exhibit increased stress responses. Consequently, an integrated analysis method integrating geometric and ACSM ILI data is proposed. This approach enhances the capability of ACSM ILI to identify pipe segments with elevated tensile stress and mitigates adverse impacts from various interference factors inherent in ACSM signals.

In accordance with Equations (2) and (3), the exported geometric and ACSM ILI data are processed to generate a new tabulated data-set. This data-set establishes the correspondence between the pipeline’s inner diameter and ACSM signal values at each inspection mileage $x_{i}$.

(2) $${R|}_{x=x_{i}}=\frac{1}{M}\cdot \sum_{m=1}^{M}{C_{m}\cdot R}_{m}$$

(3) $${U|}_{x=x_{i}}=\frac{1}{N}\cdot \sum_{n=1}^{N}{C_{n}\cdot U}_{n}$$

At each mileage point $x_{i}$, *R*_m_ represents the measured pipeline radius from each channel of the geometric ILI tool, and *U*_n_ denotes the measured voltage from each channel of the ACSM signal. M and N denote the number of sensors for the geometric and the ACSM ILI tools respectively. $C_{m}$ and $C_{n}$ denote the weighting coefficients assigned to these geometric and ACSM sensors across respective channels. In this study, we used sensors of the same model for the pipe ILI tool and performed sensors consistency screening, to ensure that all probes exhibited nearly identical sensitivity to stress and variations in inner diameters of the pipeline, despite their differing baselines. Thus the weight coefficients for all channels were uniformly set to one, and we obtain the ${R|}_{x=x_{i}}$ and ${U|}_{x=x_{i}}$ to plot the distribution profiles of pipe radius and ACSM signals along the inspection mileage.

**Figure 8:**
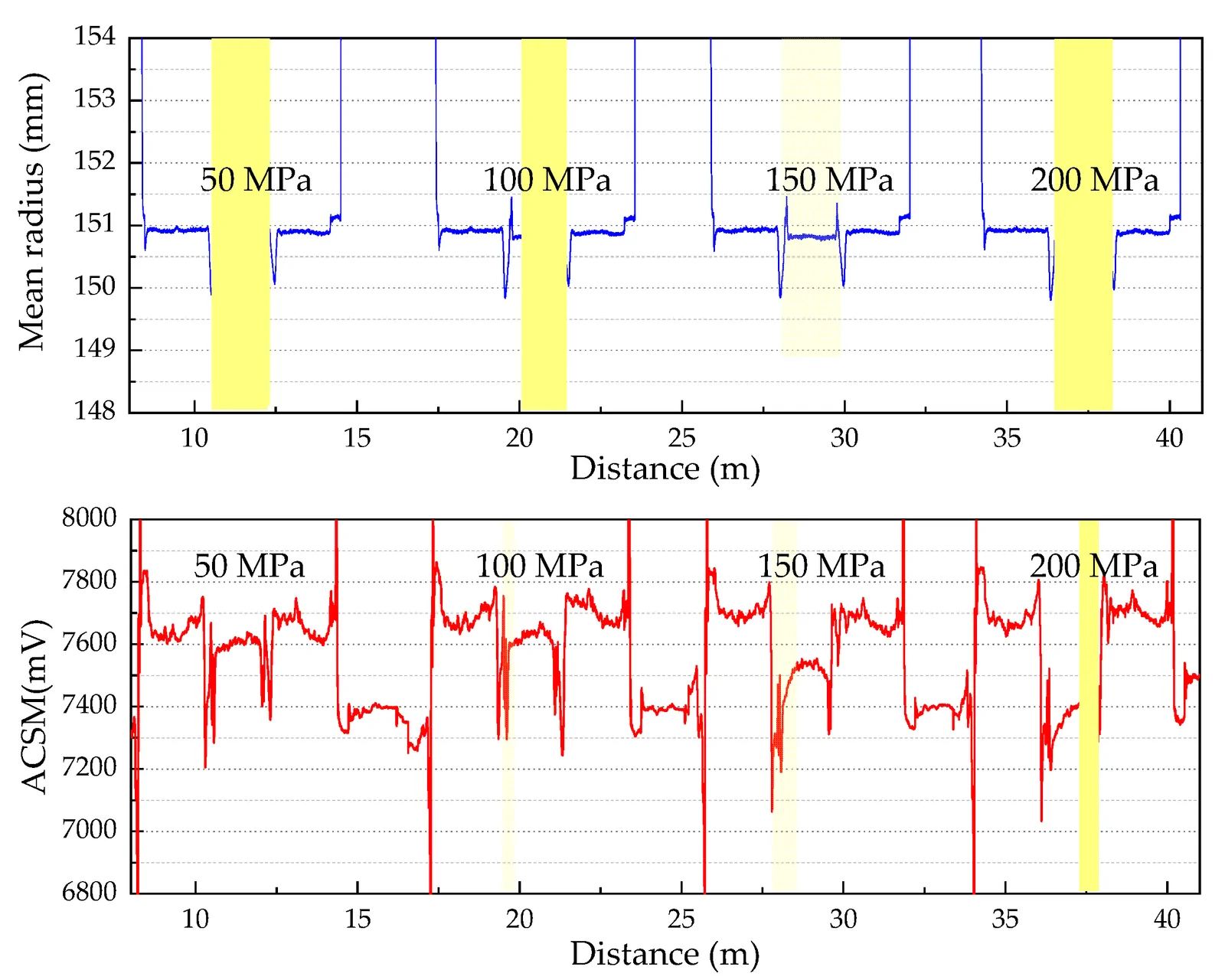
Mean value curves of radius and ACSM signal.

For the above-mentioned pull test data, we use this approach to give the curve of radius and ACSM signal for the axial tensile loaded pipe as shown in Figure 8 and Table 1.

The Radius Section of Table 1 presents the variation in the pipe’s inner diameter under different axial tensile stress levels within specific inspection mileage ranges. The reference mean radius of the pipe segment near the pig launcher is recorded as 150.92 mm. Upon applying tensile loads, the radius exhibits a slight decreasing trend. Specifically, under 50 MPa, 100 MPa, 150 MPa, and 200 MPa of axial tensile stress, the mean radii are measured as 150.86 mm, 150.81 mm, 150.80 mm, and 150.78 mm, respectively. This marginal reduction in diameter can be attributed to the Poisson’s effect, where axial elongation induces lateral contraction. Quantitatively, the maximum observed decrease in radius is merely 0.14 mm (corresponding to a change rate of approximately 0.09%). This indicates that the impact of tensile stress on the macroscopic geometry of the pipeline is extremely subtle, typically falling below the sensitivity threshold of conventional geometry inspection tools.

In stark contrast to the geometrical data, the ACSM signal demonstrates a highly sensitive response to tensile loading. The baseline signal value for the unstressed pipe section is 7644 mV. Under increasing tensile stress, the signal amplitude exhibits a significant attenuation trend:

Low to Moderate Stress Region (50–150 MPa). The signal decreases gradually from 7598 mV to 7515 mV. The incremental changes are −46 mV, −57 mV, and −84 mV, respectively. This stage likely represents the linear elastic deformation phase of the pipe material, where minor changes in magnetic permeability occur.

High Stress Region (200 MPa). A dramatic drop in the signal is observed, plummeting to 7387 mV—a single step decrement of 288 mV. This nonlinear behavior suggests that the ACSM sensors are much more sensitive for higher stress region.

Comparing the “Radius” and “ACSM Signal” Columns reveals a distinct difference in sensitivity. While the geometric radius changes are negligible (sub-millimeter scale), the ACSM signal provides a much higher resolution of mechanical state changes. The fact that the ACSM signal drops sharply at 200 MPa, whereas the radius continues to decrease linearly, implies that ACSM sensors are more effective in identifying the threats of the pipeline under high tensile stress.

### 3.3. Application of Integrated Analysis Method for an In-Service Oil Pipeline

The proposed integrated analysis method was used for the interpretation of geometric and ACSM stress ILI data acquired from an in-service D813 oil transmission pipeline. The pipeline operates at an internal pressure of approximately 4 MPa and is constructed of spiral welded pipes.

**Figure 9:**
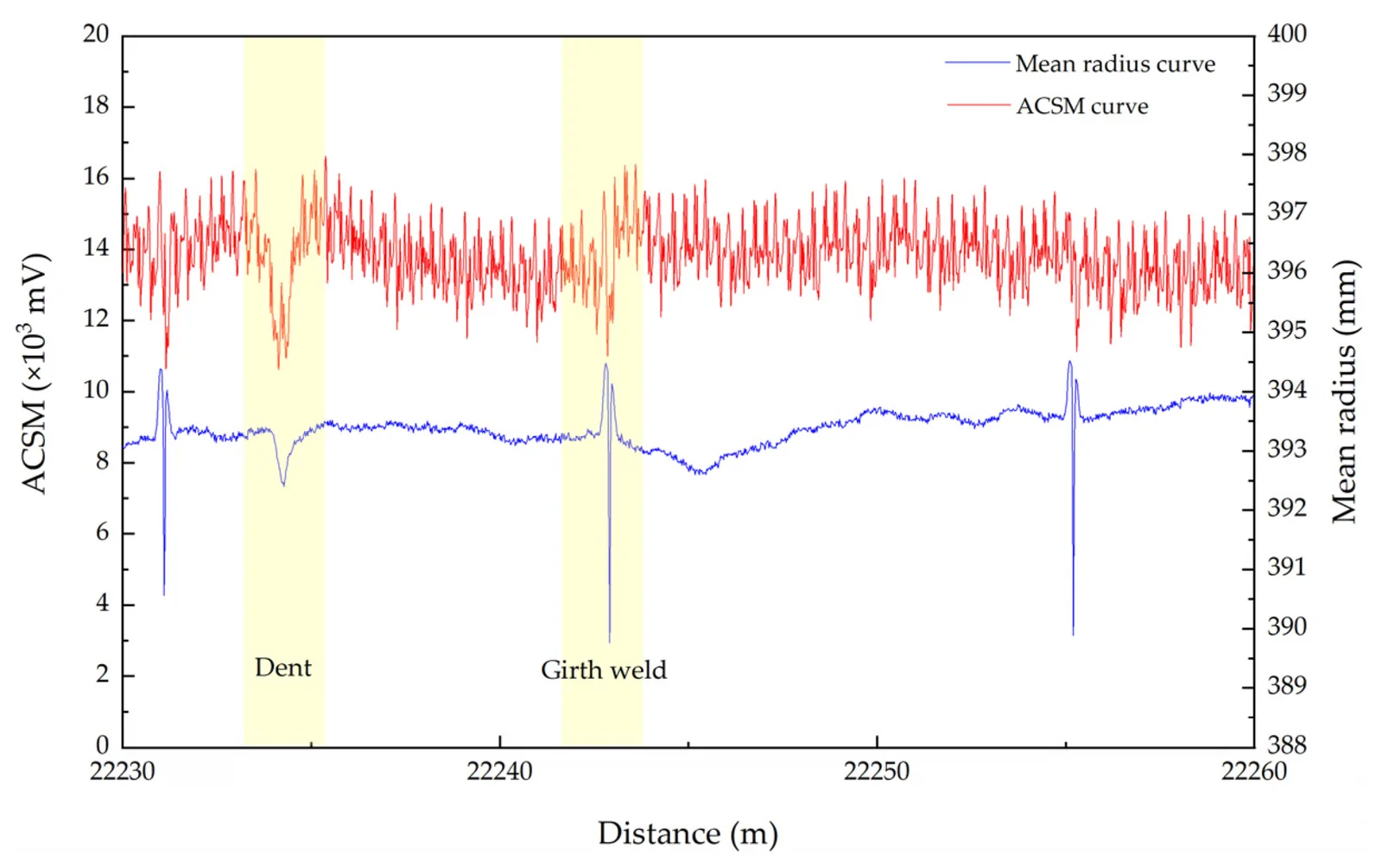
Signal characteristics of ACSM for stress concentration at pipe dent.

Figure 9 presents a comparative analysis of the ACSM signal and the mean radius profile for a pipe region with dent. The yellow shaded vertical bands highlight two critical areas of interest as the dent and girth welds. The dent is located at 22,232 m to 22,236 m, and the blue curve exhibits a pronounced trough, dropping from approximately 393.8 mm to 392.5 mm. This significant reduction in radius confirms the presence of a severe local deformation. Correspondingly, the red ACSM curve shows a distinct decrease, which corresponds to an increase in stress at the dent.

**Figure 10:**
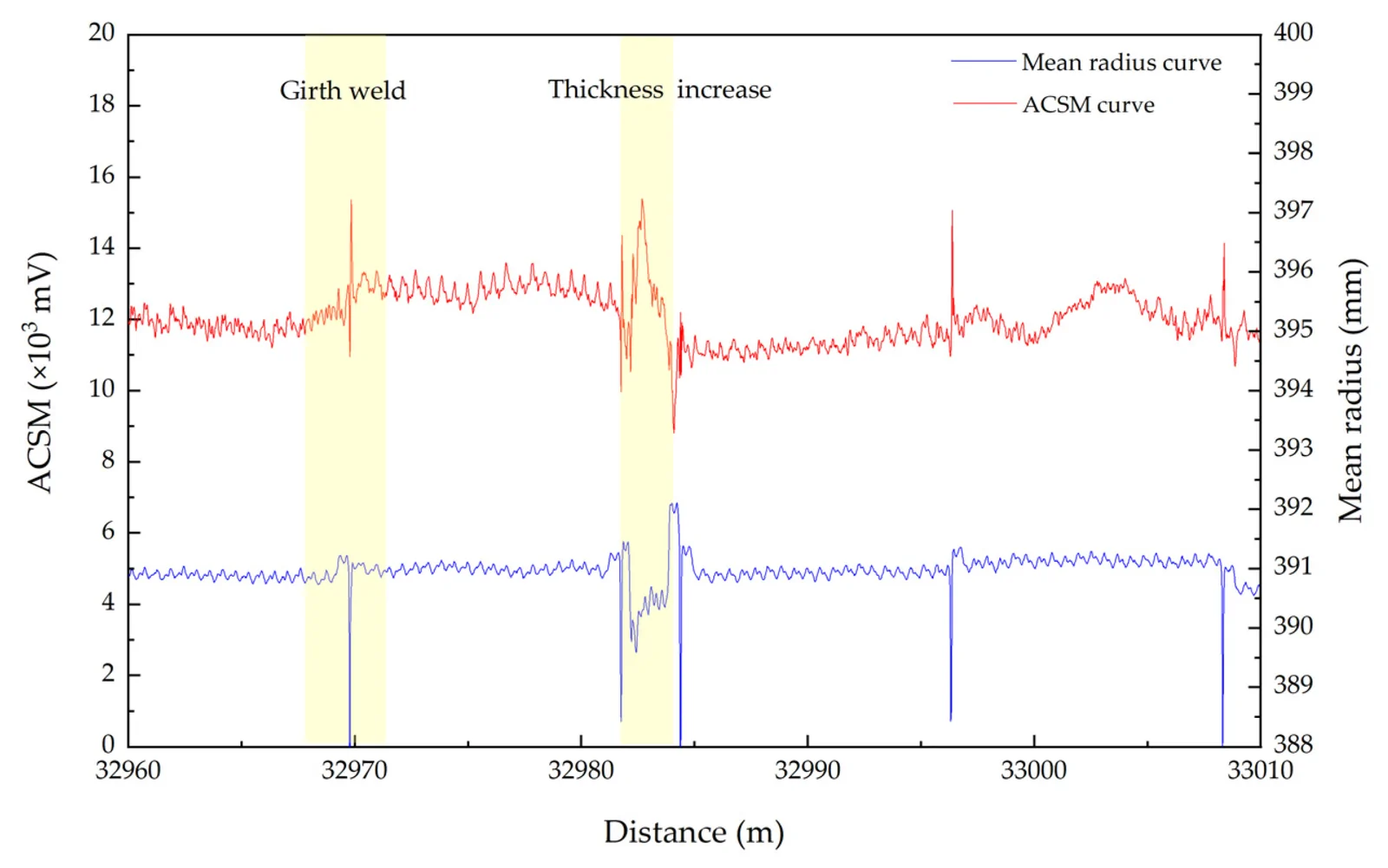
Signal characteristics of ACSM for reduced stress of pipe with increased wall thickness.

Another example corresponds to the signal signature of stress reduction induced by the localized wall thickening as shown in Figure 10. Two critical areas of interest are highlighted by yellow-shaded vertical bands as girth welds and the increase in thickness. The region with an increase in thickness is at 32,982 m to 32,988 m. The blue mean radius curve exhibits a slight, sharp downward fluctuation, indicating an increase in thickness of 0.8 mm. Correspondingly, the red ACSM curve shows a distinct, high amplitude spike, reaching a peak of approximately 15,500 mV. This significant increase reflects the reduction in pipeline stress induced by the increased wall thickness.

The synchronized behavior of the two curves validates the use of ACSM and geometric ILI data to identify stress concentration region. This approach facilitates the mitigation of adverse impacts imposed by pipeline spiral welds, mechanical noise, and other measurement interferences in fluctuations of ACSM ILI signals.

At the same time, we acknowledge certain limitations of this method. A comparison between the above mentioned in-service pipeline and the D355 test pipeline reveals that the mean signal curves of both ACSM and geometric ILI data exhibit more pronounced noise fluctuations in the former. These fluctuations likely stem from spiral welds, girth welds, bends, corrosion defects, and other features. When the ILI tools pass over such pipeline features, significant alterations occur in sensor kinematics and liftoff conditions, leading to substantial and frequent signal oscillations in both ACSM and geometric sensors. This poses considerable challenges for identifying high stress pipe segments. Consequently, further analysis of signal patterns in these sections is warranted, alongside the development of appropriate filtering and feature extraction algorithms to refine the methodology for high stress segment identification.

## 4. Conclusions

To address the challenge of identifying high stress axial tensile sections in pipelines via ACSM stress ILI, this study designed and conducted dynamic calibration tests, alongside pull tests integrating geometric and ACSM ILI sensors. Based on the experimental data and subsequent analysis, the following conclusions are drawn:

(1) Dynamic calibration tests of the ACSM sensor with pipe steel demonstrate an approximately linear correlation between axial stress and the mean value of the ACSM signals, characterized by a distinct baseline downshift under tensile loading. However, irregular high frequency signal fluctuations in the dynamic caliberation curve inhibit the performance of ACSM ILI. Since these perturbations obscure the distinction between actual stress variations and extraneous interferences, standalone ACSM data for pipe stress state interpretation remains challenging.
(2) To solve this challenge, an integrated analysis method is proposed. A pull test with geometry and ACSM ILI tools using different axial tensile loaded pipes is designed and completed. By integrating geometric deformation data, specifically correlating ACSM stress signals with concurrent reductions in pipe radius, the source of the signal can be definitively attributed to tensile deformation.
(3) Results of the pull test highlights a significant disparity in measurement sensitivity between traditional geometric inspection and the ACSM technique under varying axial tensile stresses. By applying the integrated analysis method using geometric and ACSM stress ILI data for a in-service oil pipeline, reduced radii and elevated ACSM stress signals were successfully extracted from tensioned pipe segments. Although adverse impacts were imposed by the pipeline’s spiral welds, mechanical noise, and other measurement interferences in fluctuations of ACSM ILI signals, a synchronized behavior was also demonstated between stress increase and inner radius reduction.

## Figures and Tables

**Table 1:** Change of radius and ACSM signal under the four tensile stress levels.

	Axial Tensile Stress	Range (m)	Mean Value for TensileLoaded Pipe	Mean Value of Unloaded Pipe Near Pig Launcher	Change
Radius (mm)	50 MPa	10.9~12.1	150.86	150.92	−0.06
	100 MPa	19.9~21.1	150.82	150.91	−0.09
	150 MPa	28.4~29.6	150.81	150.92	−0.11
	200 MPa	36.7~37.9	150.78	150.92	−0.14
ACSM signal (mV)	50 MPa	10.9~12.1	7598	7644	−46
	100 MPa	19.9~21.1	7622	7679	−57
	150 MPa	28.4~29.6	7515	7699	−84
	200 MPa	36.7~37.9	7387	7675	−288

## Data Availability

The original contributions presented in this study are included in the article. Further inquiries can be directed to the corresponding author.

## Abbreviations

The following abbreviations are used in this manuscript:

ILI	Inline inspection
ACSM	Alternating Current Stress Measurement
NDT	Nondestructive testing
mV	Millivolt
